# A New Definition of Pyroptosis-Related Gene Markers to Predict the Prognosis of Lung Adenocarcinoma

**DOI:** 10.1155/2021/8175003

**Published:** 2021-11-26

**Authors:** Guanran Zhang, Zhangzhe Yan

**Affiliations:** ^1^Key Laboratory for Experimental Teratology of Ministry of Education, Department of Histology & Embryology, School of Basic Medical Sciences, Shandong University, Jinan, Shandong 250012, China; ^2^Department of Surgical Oncology, Shandong Cancer Hospital and Institute, Shandong First Medical University and Shandong Academy of Medical Sciences, Jinan, Shandong 250000, China

## Abstract

Pyroptosis, the prototype of programmed cell death, is crucial to the development of multicellular organisms. Lung cancer is one of the most lethal cancers in the world. Because lung cancer progresses quickly, it is mostly found at an advanced stage, resulting in a very poor prognosis of lung cancer. At present, there is no treatment with good prognosis, but pyroptosis-based tumor therapy may be able to solve this problem. In the past few decades, it has been found that pyroptosis can affect the invasion, proliferation, and metastasis of tumor and apoptosis is an important system to resist cancer. Our study is aimed at constructing a prognostic model within pyroptosis-related genes. We developed a prognostic model by using TCGA and GEO database, and differentially expressed genes (DEGs) were identified. Five genes (NLRP1, NOD1, NLRC4, CASP9, and PLCG1) were identified to construct a prognostic model. According to the median risk score calculated by our formula, we divided patients into the high- and low-risk groups. Pyroptosis-related genes play important roles in tumor immunity and can be used to predict the prognosis of lung adenocarcinoma (LUAD).

## 1. Introduction

Lung cancer refers to the primary factor leading to deaths of cancers globally. In 2012, about 1.8 million new cases were diagnosed, of which 1.6 million died [1]. The epidemiology and prevention of lung cancer have changed dramatically in the past because the patterns of smoking are different. There has been an advance in perceiving the genetics of the cancer of the lung. Also, the role that the immune system plays in retaining the cancer of the lung. Besides, regimens of treating the cancer of lung are different. Despite perceiving the illness, treatment regimens and outcomes of cancer of the lung are improving, and survival rates remain low [2]. Lung cancer is highly heterogeneous and can occur in many various places in the bronchial system. As a result, it indicates various symptoms and patterns according to anatomical positions. Seventy percent of patients who had the cancer lung showed advanced illness [3]. The ideal intervention was surgeries for the patients who are at I-II stages of the non-small-cell lung cancer (NSCLC). The rates of survival of five years in clinics range from 77% to 92% in terms of the IA phase, 68% for the IB phase, 60% for the IIA phase, and 53% for the IIB phase. Randomized controlled trials have investigated the function of perioperative chemotherapy. Regarding the patients who are at the I phase in clinics, they undertook resection of surgeries. Or the ones that do not receive surgeries and have radiotherapy with the stereotactic body with high dose can realize controlling local tumors at a high level and toxicity at a low level [4]. Immunotherapy did not treat the cancer of the lung. However, a new intervention with great effects has come into being. Increasing interest in the immunotherapy of cancer can be seen globally [5].

The pyroptosis comes from the Greek word “ptosis” and “pyro.” The root refers to falling and fever. They are used to name a novel programmed cell death (PCD) with inflammation [6]. Pyroptosis denotes the death of cells with a pattern of inflammation, which resulted from some inflammatory bodies. It also results in the gasdermin D (GSDMD) cleavage and brings about cytokines with inactivity, including IL-1*β* and IL-18. Pyroptosis has close relationships with diabetic nephropathy and atherosclerosis. In the past few decades, cell pyroptosis has been found to have impacts on invasion, proliferation, and metastasis of the tumors. Also, it is controlled via different molecules and RNAs with noncoding [7]. In some cases, cell death is definitely good for our health, such as cancer treatment. Some existing categories of deaths of cells have been discovered, such as necrosis, apoptosis, anoikis, necrosis, pyroptosis, and autophagy. Apoptosis is a critical system to defend against cancers. It has been well researched. 2-(*α*-Naphthoyl)ethyl-trimethylammonium iodide (*α*-NETA) is a choline acetyltransferase inhibitor, inhibiting the proliferation of ovarian cancer cell lines by caspase-4-related pyroptosis [8]. In breast cancer, caspase-8-mediated pyroptosis was caused by upregulation of the expression of gasdermin C (GSDMC) [9]. However, the relationship between LUAD and pyroptosis still remains unknown. In recent years, more and more researches have been made regarding the system of molecules regarding pyroptosis of cells of tumor and mechanism of inducing tumor cell pyroptosis [10, 11]. The great effects of proinflammation of pyroptosis of cells have correlations with the control of the microenvironment of tumor with immunity [12]. It was found that pyroptosis has important functions in the defending tumor of the NK cells [13].

Based on previous studies, pyroptosis is critical for the development of tumors and the process of defending tumors. However, few studies investigate its specific functions in LUAD. TCGA database was used to establish a risk prognosis model through bioinformatics analysis, and then, the risk prognosis model was validated by the GEO database.

## 2. Materials and Methods

### 2.1. Datasets

The 594 data of RNA-seq (RNA sequencing) regarding the lung adenocarcinoma (LUAD) patients was obtained; meanwhile, the related characteristics from the database of TCGA on June 1^st^ 2021 were also obtained (http://portal.gdc.cancer.gov/repository). Data of information and RNA-seq in clinics as the cohort of the exterior validation were retrieved by the database of GEO (https://www.ncbi.nlm.nih.gov/geo/, ID: GSE72094).

### 2.2. Identification of Differentially Expressed Pyroptosis-Related Genes

33 pyroptosis-related genes were extracted by us from prior reviews [6, 14–16], and they are shown in Supplement table 1. Differentially expressed genes (DEGs) were identified between the normal tissue and the tumor. The package of “limma” was applied to divide DEGs. We use∗if*P*values less than 0.05,∗∗if*P*less than 0.01, and∗∗∗if*P*less than 0.001. The PPI system was established regarding DEGs using Search Tool for the Retrieval of Interacting Genes (STRING) version 11.0 (http://string-db.org/).

### 2.3. The Validation and Enhancement of the Prognostic Gene Paradigm concerning Pyroptosis

Cox regression analysis was used to assess the relationships between the genes and the status of survival in the cohort of TCGA. It was used to evaluate the genes concerning pyroptosis regarding the prognostic value. The cut-off *P* value was set for 0.2, and we identified 11 survival-related genes for analysis to prevent omissions. Then, the paradigm of regression of the least absolute shrinkage and selection operator (LASSO) Cox (R package “glmnet”) was used to filter the relevant genes. It was also applied to establish the prognostic paradigm. The last step was the retention of the five genes and the coefficients. The penalty parameter (*λ*) got clarified using the lowest criteria. The score of risks was analyzed when the data of expression of TCGA was standardized and centralized (using the “scale” function in *R*). The formula of the score of risks was risk score = ∑_*i*_^5^*Xi*∗*Yi* (*X* stands for coefficients and *Y* stands for the level of expression of the genes). Following the median scores of risk, the patients with the cancer of the lung were grouped into the high-risk and low-risk groups. Next, the OS duration was the comparison of the two subcategories using the analysis of Kaplan–Meier. PCA, according to five signatures of gene, was conducted using the function of “prcomp” in the R package using the function of “stats.” Also, the “survival,” “timeROC,” and “survminer” R packages analyzed the curve of ROC. A cohort of LUAD was retrieved from the database of GEO (GSE72094) for validating the studies. The function of “scale” was applied to normalize the level of expression of genes concerning pyroptosis. The score of risks was examined using the same formula in the cohort of TCGA. The patients who had GSE72094 were grouped into the group with high risks and the group with low risks following the risk medians. The group with high risks and the group with low risks were compared for the validation of the prognostic paradigm.

### 2.4. Analyzing the Independent Prognostic of the Score of the Risk

We extracted the clinical information of patients in the TCGA cohort and GEO cohort and we employed models of univariable and multivariable Cox regression to analyze the independent prognostic of the score of the risk.

### 2.5. Functional Enrichment Analysis of the DEGs between the Low-Risk Groups and High-Risk Groups

According to the median score, we stratified analyzing and enriching the functions of the DEGs between the group with high risks and the group with low risks of the patients who had LUAD in the cohort of TCGA further into two groups. Next, based on specific criteria (FDR less than 0.05 and |log_2_FC| greater than or equal to one), the DEGs between the two groups with greater risks and the group with weaker risks were screened. We performed Gene Ontology (GO) enrichment analysis and Kyoto Encyclopedia of Genes and Genomes (KEGG) pathway analyses based on the DEGs by applying the “clusterProfile” package. The package of “gsva” was used to perform the single-sample gene set enrichment analysis (ssGSEA) for examining the scores to infiltrate immunity cells and assessing the patterns of the pathways regarding immunity.

### 2.6. Statistical Analysis

**Figure 1:**
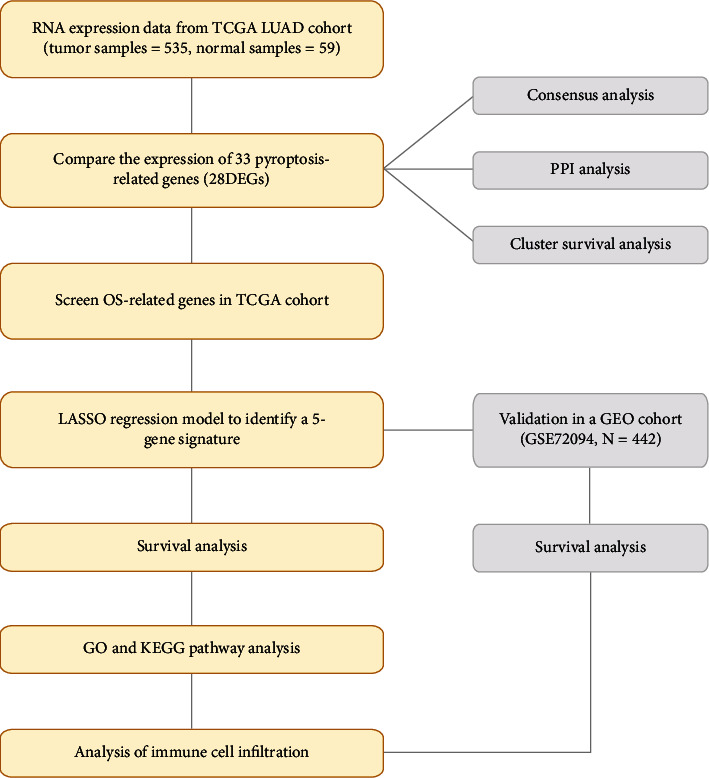
Study workflow diagram.

We applied single-factor analysis of variance for the comparison of levels of expression of genes between tissues of LUAD and normal lung tissues, with the Pearson chi-square test being used for the comparison of categorical variables. The Kaplan–Meier method was used combining a log-rank test with two sides for comparisons regarding OS of the patients among the groups. Multivariate and univariate Cox regression models evaluated the risk model's independent prognostic value. We employed the Mann-Whitney test while we compared the immune pathway activation and immune cell infiltration in the 2 groups. We accomplished all statistical analyses by using R software version (4.0.2).  Figure 1 indicates the flow diagram.

## 3. Results

### 3.1. Determining the DEGs between the Tissues of Tumors and the Common Tissues

**Figure 2:**
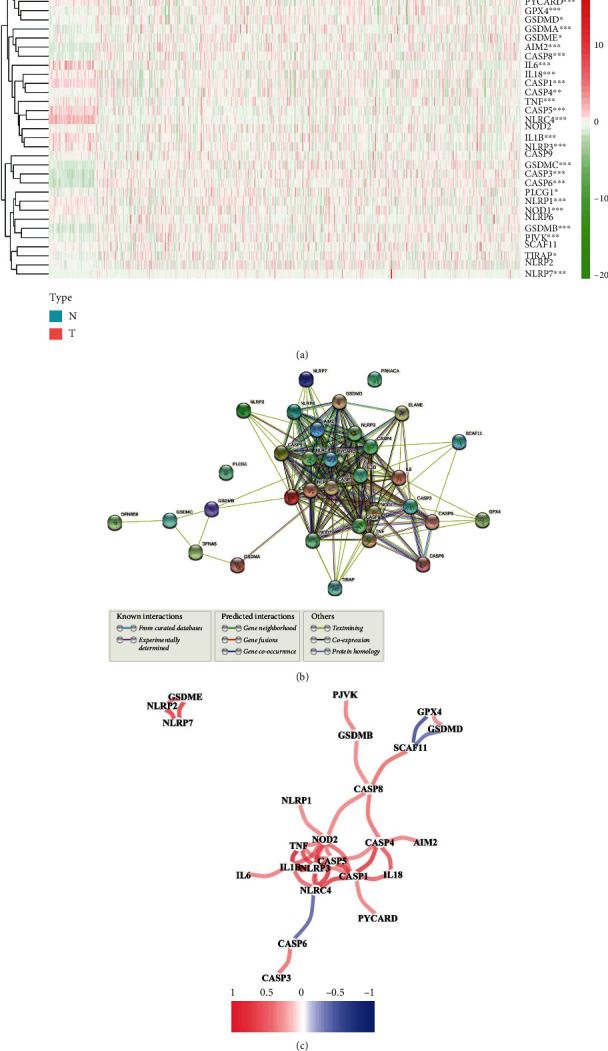
Expression and interaction of 33 pyroptosis-related genes. (a) Heatmap of the genes concerning pyroptosis between the tissues of the tumor (T, red) and the common tissues (N, blue) (red: level of expression at a high level; green: level of expression at a low level). *P* values: ^∗^*P* less than 0.05, ^∗∗^*P* less than 0.01, and ^∗∗∗^*P* less than 0.001. (b) The network of protein-protein interaction (PPI) indicated the interaction of genes concerning the pyroptosis (the interaction score = 0.4). (c) Relevant network of the genes concerning pyroptosis (blue line: negative relationships; red line: positive relationships; the depth of the color shows the relationship intensity).

A total of 33 genes were found as pyroptosis-related genes. The information was regarding 59 common tissues and 535 tumor tissues. We used the package of “limma” to find DEGs, and the threshold value was 0.05. Twenty-eight genes were found to be differentially expressed genes (DEGs). According to the heatmap, we found that 12 genes (PRKACA, PYCARD, IL6, IL18, CASP1, TNF, CASP5, NLRC4, IL1B, NLRP3, NLRP1, and NOD1) decreased. Also, other 16 genes (ELANE, GPX4, GSDMD, GSDMA, GSDME, AIM2, CASP8, CASP4, GSDMC, CASP3, CASP6, PLCG1, GSDMB, PJVK, TIRAP, and NLRP7) got improved in the group of the tumor. The levels of RNA regarding the genes are indicated to be heatmaps, as shown in Figure 2(a) (red: level of expression at a high level; green: level of expression a low level). An analysis of protein-protein interaction (PPI) was adopted to further explore the communications and interactions of these pyroptosis-related genes, as shown in Figure 2(b). We set 0.4 (the interaction score) as the lowest score of interaction needed by the analysis of PPI. Next, PYCARD, CASP1, IL1B, IL18, TNF, NLRC4, AIM2, and CASP8 were hub genes by Cytoscape software (Supplement table 2). All of them were DEGs of tissues between tumor and common tissues. According to Figure 2(c), there is a network of relationships of the entire genes concerning pyroptosis (blue: negative relationships; red: positive relationships).

### 3.2. The Classification of the Tumors following the DEGs

**Figure 3:**
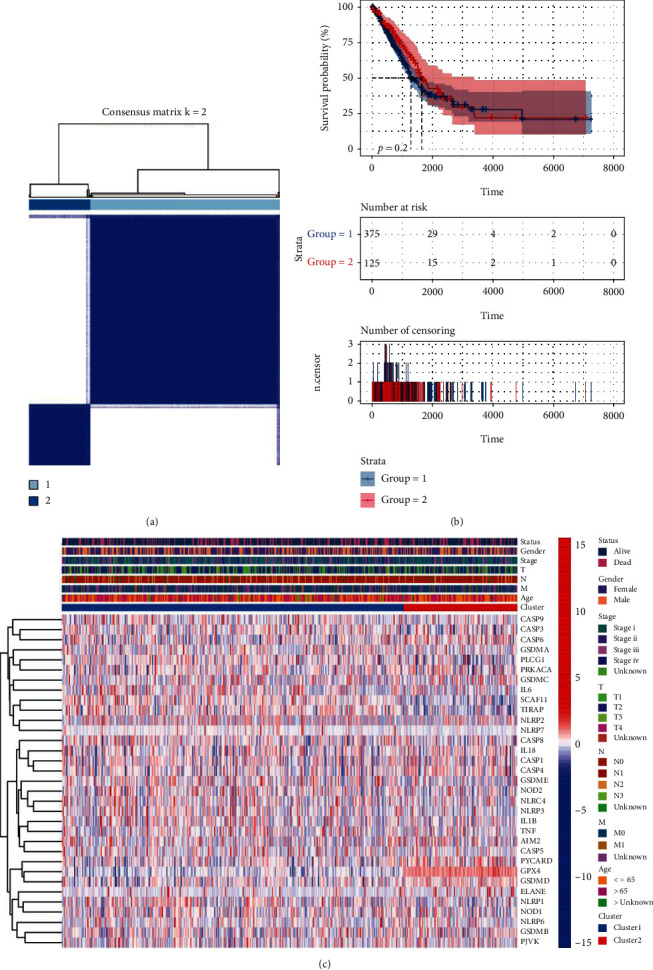
The tumors were classified according to the pyroptosis-related DEGs. (a) 535 patients were grouped into two subgroups following the consensus matrix (*k* = 2). (b) OS curve of Kaplan-Meier regarding the two clusters. (c) Characters of pathology in clinics and heatmap regarding the two subgroups sorted according to the DEGs (T: primary tumor; T1: diameter of tumor ≤ 3 cm and no peripheral metastasis; T2: 3 cm ≤ diameter of tumor ≤ 5 cm or spreading to hilum with atelectasis; T3: 5 cm ≤ diameter of tumor ≤ 7 cm; T4: tumor ≥ 7 cm; N: lymph node; N0: no lymph node metastasis; N1: peribronchial or ipsilateral hilar lymph node metastasis; N2: ipsilateral mediastinal or subcarinal lymph node metastasis; N3: contralateral hilar, mediastinal or scalenus, and supraclavicular lymph node metastasis; M: distant metastasis; M0: no distant metastasis; M1: has distant metastasis).

An analysis of clustering of consensus was adopted with the entire 535 patients in the cohort of TCGA for the investigation of the relations between the expression of the 28 DEGs concerning pyroptosis and the subtypes of LUAD. The variable of clustering (*k*) was raised from two to nine. The results show that while *k* = 2, the intergroup relationships were at the lowest level and the intragroup relationships had the highest level. It showed the 535 patients would be well grouped into two categories according to these 28 DEGs (as shown in Figure 3(a)). There were 500 patients with complete survival data. Also, the overall survival (OS) time was compared between them without a significance (with *P* being 0.2, as shown in Figure 3(b)). According to the heatmap, the characteristics in clinics and the profile of the expression of genes consisted of the ageing degrees (greater than 65 years or less than, equal to 65 years or unknown), gender (male or female), stage (i, ii, iii, iv, or unknown), and TNM classification survival status (dead or alive). However, almost no difference was found in the characteristics in clinics between the two groups (as shown in Figure 3(c)).

### 3.3. Enhancing a Paradigm of the Prognostic Gene in the Cohort of TCGA

**Figure 4:**
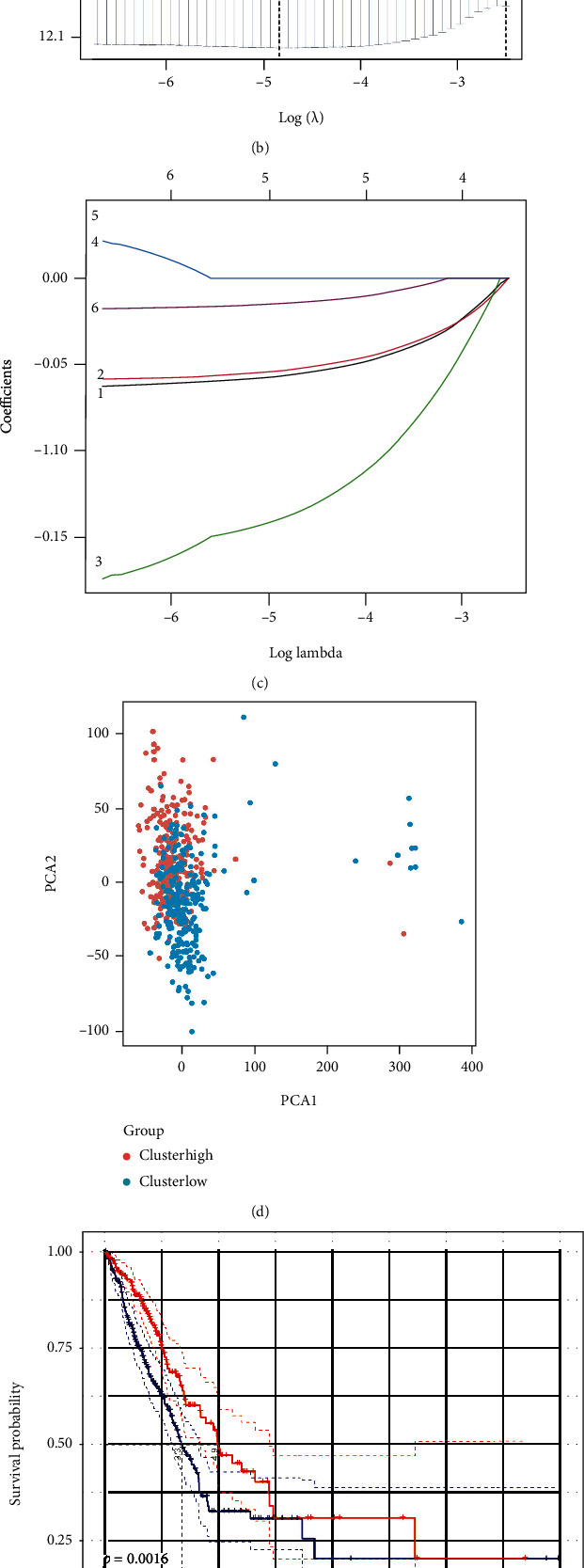
Establishing signatures of risk in the queues of the TCGA. (a) The univariate Cox regression analysis reported that OS of all the 33 genes about pyroptosis and eleven genes had *P* less than 0.2. (b) Cross-validation for modifying selecting the parameter. (c) The regression of LASSO of six OS-related genes. (d) LUAD PCA plot following the score of the risk. (e) The curve of Kaplan-Meier of the OS in the group at high risk and the group at a low risk. (f) According to the curves of ROC, the efficiency of the score of the risk could be predicted. (g) The status of survival of the patients (on the right hand of the dotted line: the group at high risk; on the left of the dotted line: the group at low risk).

The patients who had complete survival information were matched with a total of 500 LUAD samples. We used univariate Cox regression to screen the genes. Eleven genes (NLRC4, NLRP1, NOD1, NOD2, NLRP3, PRKACA, PLCG1, TNF, CASP1, CASP9, and CASP6) were found to fulfil the requirements with *P* value less than 0.2. They were kept for follow-up investigations. Two genes of them (CASP6 and CASP9) had relationships with the improved risk with HRs larger than 1. However, the other nine genes (NLRC4, NLRP1, NOD1, NOD2, NLRP3, PRKACA, PLCG1, TNF, and CASP1) belonged to protective genes with HRs less than 1 (Figure 4(a)). An analysis of the least absolute shrinkage and selection operator (LASSO) revealed Cox regression was adopted. A pattern of five genes was established following the ideal *λ* score (as shown in Figures 4(b) and 4(c)). The value regarding the risk was as follows: (−0.057∗NLRP1 exp.) plus (−0.139∗NLRC4 exp.) plus (−0.054∗NOD1 exp.) plus (0.034∗CASP9 exp.) plus (−0.015∗PLCG1 exp.). We divided 500 patients equally into subgroups at low risk and at high risk (as shown in Figure 4(g)) following the results of median values. According to principal component analysis (PCA), the patients who were at various risks were greatly grouped into two parts (as shown in Figure 4(d)). The mortality number of the patients in the group at high risk was more and got a shorter time for survival compared to the ones in the group at low risk (as shown in Figure 4(g), on the right of the dotted line). The OS time had a significant difference. It was retrieved between the group at high risk and the group at low risk (*P* = 0.0016, as shown in Figure 4(e)). The time-dependent analysis of receiver operating characteristic (ROC) was used to evaluate the specificity and sensitivity of the prognostic model. Also, the area under the ROC curve (AUC) was 0.67 for ten-year, 0.62 for eight-year, 0.62 for five-year, and 0.59 for three-year, as well as 0.54 for one-year survival (as shown in Figure 4(f)).

### 3.4. Exterior Validation of the Pattern of the Risk

**Figure 5:**
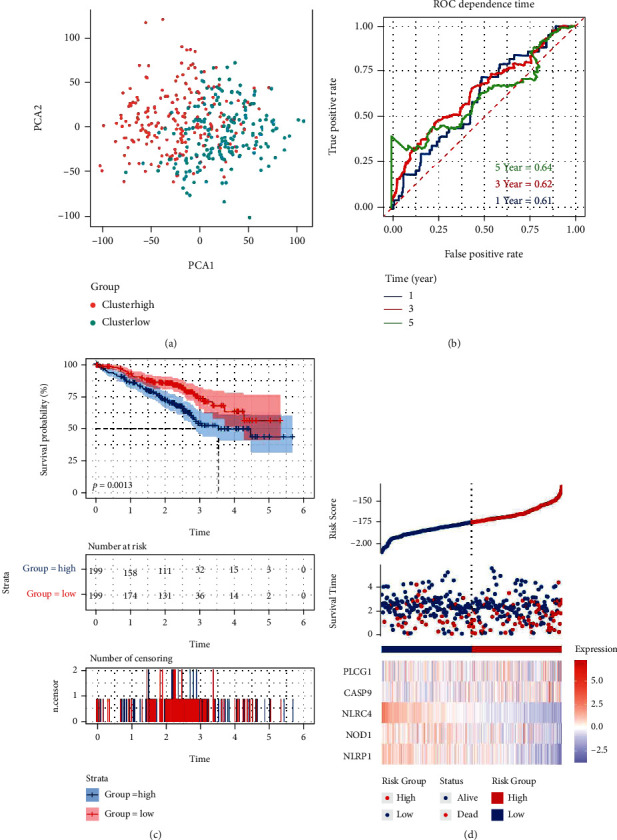
Validation of the risk paradigm in the cohort of GEO. (a) LUAD PCA plot (b) LUAD ROC curves of the time dependence. (c) The patient distribution in the cohort of GEO following median values of the risk in the cohort of GEO. Kaplan-Meier curve compared the OS in the group at low risk and the group at high risk. (d) The status of survival of the patient (on the right-hand side of the dotted line: the group at high risk; on the left-hand side of the dotted line: the group at low risk).

Four hundred and forty-two patients who had LUAD from the GSE72094 cohort of Gene Expression Omnibus (GEO) were regarded as the set of validation. The data of the expression of genes were generalized using the “Scale” function before further analysis. One hundred and ninety-nine patients in the cohort of GEO were identified as the group at low risk. The patients were grouped into the group at high risk following the median value of the risk (as shown in Figure 5(d)). The PCA indicated perfect separation between the group at low risk and the group at high risk (as shown in Figure 5(a)). The patients in the subgroup at low risk (on the left of the dotted line, according to Figure 5(d)), lived longer, compared to the ones in the subgroup at high risk. What is more, according to the analysis of Kaplan-Meier, an explicit significance was found in the rate of survival between these groups (*P* = 0.0013, as shown in Figure 5(c)). Our model was found to have ideal efficacy for prediction (AUC = 0.64 for five-year and 0.62 for three-year, as well as 0.61 for one-year survival) with analyzing the curve of ROC in the cohort of GEO (Figure 5(b)).

### 3.5. Independent Value of the Prognostic regarding the Model of Risk

**Figure 6:**
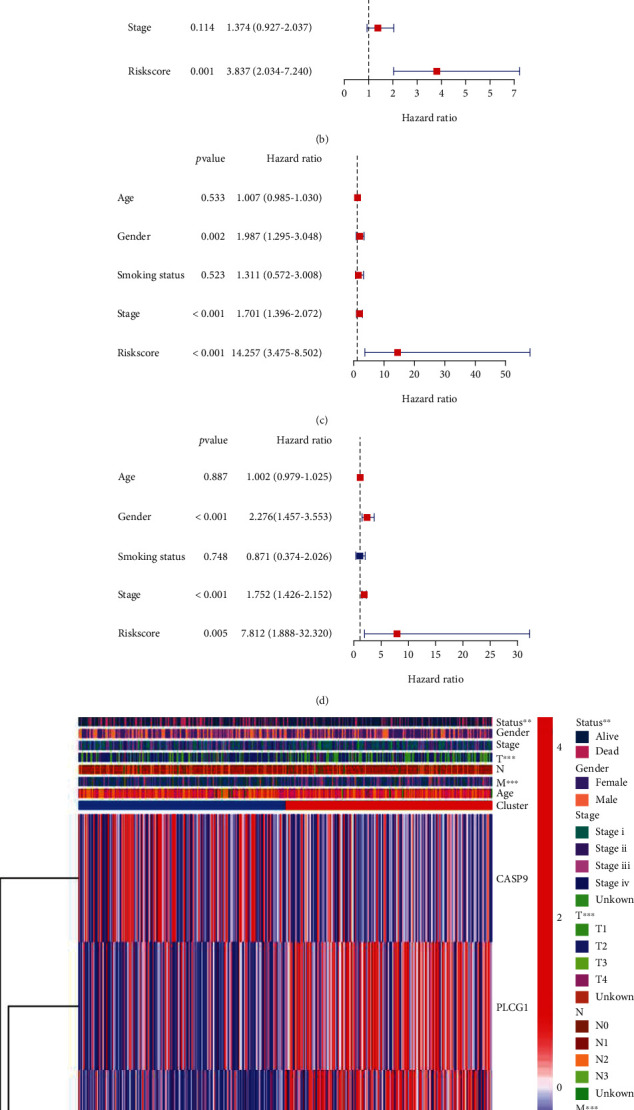
Univariate Cox regression analysis and multivariate Cox regression analysis of the value of the risk. (a) Univariate analysis of the cohort of TCGA. (b) Multivariate analysis of the sample of TCGA. (c) Univariate analysis of the cohort of GEO. (d) Multivariate analysis of the sample of GEO. (e) The heatmap (red: presentation at a great level; green: expression at a weak level), indicating the relationships between the risk groups and the clinicopathological characteristics (^∗^*P* less than 0.05).

Univariate Cox regression analyses and multivariable Cox regression analyses were used, and the risk scores of the gene model would be regarded as the independent factor of the prognostic. Firstly, the score of the risk would be regarded as an independent factor showing low survival within the two cohorts, including GEO and TCGA (HR: 14.257, 95% CI: 3.475–58.502; HR: 3.731, 95% CI: 2.071–6.724, as shown in Figures 6(a) and 6(c)). To make it evident, the multivariate analysis was adopted following the adjustment of other confounding factors. The score of the risk can be defined as a prognostic factor (HR: 3.837, 95% CI: 2.034–7.240; HR: 7.812, 95% CI: 1.888–32.320, shown Figures 6(b) and 6(d)) for patients with LUAD in the cohort of TCGA and GEO. A heatmap was constructed from characteristics in clinics regarding the cohort of TCGA (as shown in Figure 6(e)). M, T, and gender of the patients and the status of survival were found to have diverse distributions between the subgroup at a low risk and the subgroup at a high risk (*P* less than 0.05).

### 3.6. Functional Analyses and Immune Analyses following the Risk Model

The R package of “limma” was applied to extract the DEGs via the application of the criteria FDR less than 0.05 and |log_2_FC| greater than or equal to one, for follow-up investigation of the significances in the pathways and functions of the genes between the subcategories sorted following the model of the risk. The enrichment analysis of Gene Ontology (GO) and the analysis of pathway of Kyoto Encyclopedia of Genes and Genomes (KEGG) were adopted following the DEGs. In the TCGA cohort, 3852 DEGs between the low- and high-risk groups were identified. Among them, 1916 genes were upregulated in the high-risk group, while the other 1936 genes were downregulated (Supplement table 3).

**Figure 7:**
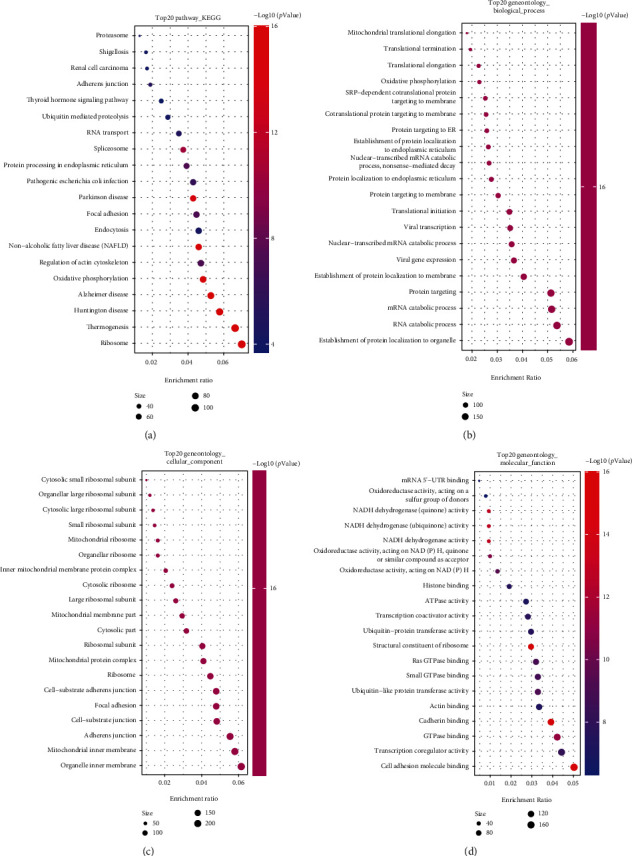
Based on the functional analysis of DEGs between the two categories of the risk in TCGA. (a) The pathway bubble diagram of KEGG. (b–d) Bubble graph of the enrichment of GO (the more genes enriched, the larger bubble; the more obvious the differences were, the more depth the color red is).

**Figure 8:**
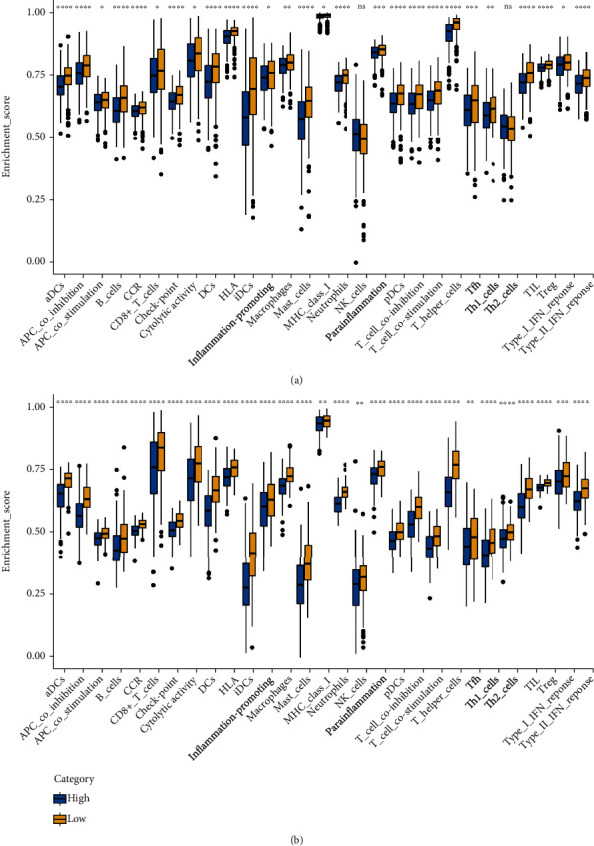
The values of ssGSEA regarding cells of immunity and pathways of the immunity were compared. (a) Sixteen immune cells and 13 pathways about immunity were compared between the low-risk group (marked in yellow) and the high-risk group (marked in blue) among TCGA. (b) Sixteen immune cells and 13 pathways about immunity were compared between the low-risk group (marked in yellow) and the high-risk group (marked in blue) in GEO cohort. ^∗^*P* less than 0.05; ^∗∗^*P* less than 0.01; ^∗∗∗^*P* less than 0.001.

According to the results, the DEGs had relationships about ribosome, organelle inner membrane, cell adhesion molecule binding, and establishment of protein localization to organelle (Figures 7(a)–7(d)). The functional analyses showed the differences between the patterns of immunity of the subcategories. There are enrichment scores of the patterns of 13 pathways concerning immunity and 16 sorts of the cells of immunity between the group at low risk and the group at high risk within GEO and TCGA using the single-sample gene set enrichment analysis (ssGSEA). In the cohort of TCGA (as shown in Figure 8(a)), the subgroup at a high risk mostly had lower infiltration levels of immune cells compared to the subgroup at a low risk. All 13 pathways of immunity indicated weaker patterns in the group at risk at a high level, compared to the one at low risk in the cohort of TCGA (as shown in Figure 8(a)). Evaluating the immune status in the cohort of GEO led to similar findings (Figure 8(b)).

## 4. Discussion

In our study, we identified a prognostic models of five pyroptosis-related genes, and confirmed the validity and practicability of the model, which can provide a lot of guidance for clinical application.

To further understand our prognostic model, we searched the function of those genes. PLCG1, known as phospholipase C, gamma 1. The protein encoded by this gene catalyzes the formation of inositol 1,4,5-trisphosphate and diacylglycerol from phosphatidylinositol 4,5-bisphosphate. This reaction uses calcium as a cofactor and plays an important role in the intracellular transduction of receptor-mediated tyrosine kinase activators. NOD1 is known as nucleotide-binding oligomerization domain containing 1. This gene encodes a member of the NOD (nucleotide-binding oligomerization domain) family. This member is a cytosolic protein. It is said that this gene initiates inflammation. NLRC4 encodes a member of the caspase recruitment domain-containing NLR family. Family members play essential roles in innate immune response to a wide range of pathogenic organisms, tissue damage, and other cellular stresses. NLRP1 is involved in the composition of inflammasome, and the activation of inflammasome can also induce pyroptosis. It has been identified that many inflammasomes are involved in host defense response against a variety of pathogens, and pathogens have evolved various corresponding mechanisms to inhibit inflammasome activation.CASP9 is the only gene with HR>1; this gene encodes a member of the cysteine-aspartic acid protease (caspase) family. Sequential activation of caspases plays a central role in the execution-phase of cell apoptosis. The more expression of CASP9, the more apoptosis occurred in those cells.

Pyroptosis, the prototype of programmed cell death, is crucial to the development of multicellular organisms. Pyroptosis was characterized by the activation of the caspase family of cysteine proteases. Activate the caspase to receive external or internal apoptosis cues, and then activate the executioner caspase to initiate the death program [17–22]. A large number of studies have shown that pyroptosis is closely related to the occurrence and development of cancer and other diseases. A further study on the mechanism of pyroptosis and its relationship with tumors will broaden our understanding of tumors and provide a new perspective for the prevention and treatment of tumors [23–25]. Our study is aimed at finding some pyroptosis-related genes which can be constructed as a prognostic model.

Our study has great clinical significance. We established the prognosis model of five genes related to pyroptosis. These five genes can be used to diagnose patients, and the prognosis model can be used to predict the prognosis of patients. If we can establish relevant targeted drugs to target these five genes in the future, it may open up new ideas for cancer treatment and create great clinical value. Secondly, we explain the relationship between different immune subtypes and prognosis models. The poor immune infiltration in the high-risk group may indicate that tumor cells are not recognized and attacked by immune cells, which leads to poor prognosis. We can use immunoactivation therapy for the high-risk group, so as to improve the tumor infiltration degree of immune microenvironment in the high-risk group. We can also take advantage of the high level of immune infiltration in the low-risk group and use immunotherapy to improve the interests of patients to a new height.

However, we still have some shortcomings and limitations. For example, the innovation of bioinformatics analysis is not abundant. The sample size selected is only from LUAD, not LC. These may lead to a narrow range of clinical indications.

Although our study has some limitations, our study clearly provides a five-gene prognosis model, which can be used as an independent prognostic factor to predict the survival of patients and can also be used for immunotyping of lung cancer patients. This provides a great guiding value for clinical diagnosis and treatment.

## Data Availability

If necessary, you can contact the corresponding author for data.

## Supplementary Materials

Supplementary Materials A small part of the article data in supplementary materials.

## References

[1] Nasim F., Sabath B. F., Eapen G. A. (2019). Lung cancer. *The Medical Clinics of North America*.

[2] Bade B. C., Dela Cruz C. S. (2020). Lung cancer 2020: epidemiology, etiology, and prevention. *Clinics in Chest Medicine*.

[3] Lemjabbar-Alaoui H., Hassan O. U., Yang Y. W., Buchanan P. (2015). Lung cancer: biology and treatment options. *Biochimica et Biophysica Acta (BBA)-Reviews on Cancer*.

[4] Hirsch F. R., Scagliotti G. V., Mulshine J. L. (2017). Lung cancer: current therapies and new targeted treatments. *Lancet*.

[5] Steven A., Fisher S. A., Robinson B. W. (2016). Immunotherapy for lung cancer. *Respirology*.

[6] Xia X., Wang X., Cheng Z. (2019). The role of pyroptosis in cancer: pro-cancer or pro-"host"?. *Cell Death Discovery*.

[7] Fang Y., Tian S., Pan Y. (2020). Pyroptosis: a new frontier in cancer. *Biomedicine & Pharmacotherapy*.

[8] Qiao L., Wu X., Zhang J. (2019). *α*‐NETA induces pyroptosis of epithelial ovarian cancer cells through the GSDMD/caspase-4 pathway. *The FASEB Journal*.

[9] Hou J., Zhao R., Xia W. (2020). Author correction: PD-L1-mediated gasdermin C expression switches apoptosis to pyroptosis in cancer cells and facilitates tumour necrosis. *Nature Cell Biology*.

[10] Ye Y., Dai Q., Qi H. (2021). A novel defined pyroptosis-related gene signature for predicting the prognosis of ovarian cancer. *Cell Death Discovery*.

[11] Zhang Z., Zhang Y., Xia S. (2020). Gasdermin E suppresses tumour growth by activating anti-tumour immunity. *Nature*.

[12] Shi J., Gao W., Shao F. (2017). Pyroptosis: gasdermin-mediated programmed necrotic cell death. *Trends in Biochemical Sciences*.

[13] Ruan J., Wang S., Wang J. (2020). Mechanism and regulation of pyroptosis-mediated in cancer cell death. *Chemico-Biological Interactions*.

[14] Karki R., Kanneganti T. (2019). Diverging inflammasome signals in tumorigenesis and potential targeting. *Nature Reviews. Cancer*.

[15] Wang B., Yin Q. (2017). AIM2 inflammasome activation and regulation: A structural perspective. *Journal of Structural Biology*.

[16] Man S., Kanneganti T. (2015). Regulation of inflammasome activation. *Immunological Reviews*.

[17] Humphries F., Shmuel-Galia L., Ketelut-Carneiro N. (2020). Succination inactivates gasdermin D and blocks pyroptosis. *Science*.

[18] Tang R., Xu J., Zhang B. (2020). Ferroptosis, necroptosis, and pyroptosis in anticancer immunity. *Journal of Hematology & Oncology*.

[19] Vande Walle L., Lamkanfi M. (2016). Pyroptosis. *Current Biology*.

[20] Hu L., Chen M., Chen X. (2020). Chemotherapy-induced pyroptosis is mediated by BAK/BAX-caspase-3-GSDME pathway and inhibited by 2-bromopalmitate. *Cell Death & Disease*.

[21] Zhang Y., Liu X., Bai X. (2018). Melatonin prevents endothelial cell pyroptosis via regulation of long noncoding RNA MEG3/miR-223/NLRP3 axis. *Journal of Pineal Research*.

[22] Tavakoli Dargani Z., Singla D. K. (2019). Embryonic stem cell-derived exosomes inhibit doxorubicin-induced TLR4-NLRP3-mediated cell death-pyroptosis. *American Journal of Physiology-Heart and Circulatory Physiology*.

[23] Wang Y., Yin B., Li D., Wang G., Han X., Sun X. (2018). GSDME mediates caspase-3-dependent pyroptosis in gastric cancer. *Biochemical and Biophysical Research Communications*.

[24] Zhou C. B., Fang J. Y. (2019). The role of pyroptosis in gastrointestinal cancer and immune responses to intestinal microbial infection. *Biochimica Et Biophysica Acta Reviews on Cancer*.

[25] Zhang C. C., Li C. G., Wang Y. F. (2019). Chemotherapeutic paclitaxel and cisplatin differentially induce pyroptosis in A549 lung cancer cells via caspase-3/GSDME activation. *Apoptosis*.

