# Supplementary material for: A New Definition of Pyroptosis-Related Gene Markers to Predict the Prognosis of Lung Adenocarcinoma
Source: Biomed Res Int. 2021 Nov 26;2021:8175003. doi: 10.1155/2021/8175003 (PMC8642010; doi:10.1155/2021/8175003)
Supplement: Supplementary Materials — A small part of the article data in supplementary materials. [file 8175003.f1.zip › citation.pdf]

Supplement table 1: 33 pyroptosis-related genes
Supplement table 2: top 8 hub genes in protein-protein interaction
Supplement table 3: 1916 up-regulated genes and 1936 down-regulated genes
