# Supplementary material for: A New Definition of Pyroptosis-Related Gene Markers to Predict the Prognosis of Lung Adenocarcinoma
Source: Biomed Res Int. 2021 Nov 26;2021:8175003. doi: 10.1155/2021/8175003 (PMC8642010; doi:10.1155/2021/8175003)
Supplement: Supplementary Materials — A small part of the article data in supplementary materials. [file 8175003.f1.zip › Supplement table 1.pdf]

**Supplement table 1** 33 pyroptosis-related genes

| Genes | Full—names |
|---|---|
| AIM2 | Absent in melanoma 2 |
| CASP1 | cysteine—aspartic acid protease—1 |
| CASP3 | cysteine—aspartic acid protease—3 |
| CASP4 | cysteine—aspartic acid protease—4 |
| CASP5 | cysteine—aspartic acid protease—5 |
| CASP6 | cysteine—aspartic acid protease—6 |
| CASP8 | cysteine—aspartic acid protease—8 |
| CASP9 | cysteine—aspartic acid protease—9 |
| ELANE | elastase, neutrophil expressed |
| GPX4 | glutathione peroxidase 4 |
| GSDMA | gasdermin A |
| GSDMB | gasdermin B |
| GSDMC | gasdermin C |
| GSDMD | gasdermin D |
| GSDME | gasdermin E |
| IL18 | interleukin 18 |
| IL1B | interleukin 1 beta |
| IL6 | interleukin 6 |
| NLRC4 | NLR family CARD domain containing 4 |
| NLRP1 | NLR family pyrin domain containing 1 |
| NLRP2 | NLR family pyrin domain containing 2 |
| NLRP3 | NLR family pyrin domain containing 3 |
| NLRP6 | NLR family pyrin domain containing 6 |
| NLRP7 | NLR family pyrin domain containing 7 |
| NOD1 | nucleotide binding oligomerization domain containing 1 |
| NOD2 | nucleotide binding oligomerization domain containing 2 |
| PJVK | pejvakin/deafness, autosomal recessive 59 |
| PLCG1 | phospholipase C gamma 1 |
| PRKACA | protein kinase cAMP—activated catalytic subunit alpha |
| PYCARD | PYD and CARD domain containing |
| SCAF11 | SR—related CTD associated factor 11 |
| TIRAP | TIR domain containing adaptor protein |
| TNF | tumor necrosis factor |
