# Supplementary material for: A New Definition of Pyroptosis-Related Gene Markers to Predict the Prognosis of Lung Adenocarcinoma
Source: Biomed Res Int. 2021 Nov 26;2021:8175003. doi: 10.1155/2021/8175003 (PMC8642010; doi:10.1155/2021/8175003)
Supplement: Supplementary Materials — A small part of the article data in supplementary materials. [file 8175003.f1.zip › Supplement table 2.pdf]

Supplement table 2 Top 8 in network string-ppi_interactions.tsv ranked by Degree method

Rank	Name	Score
1	PYCARD	23
1	CASP1	23
3	IL1B	22
3	IL18	22
5	TNF	20
6	NLRC4	19
7	AIM2	18
7	CASP8	18
